# Decreased serum levels of thrombospondin‐1 in female depressed patients

**DOI:** 10.1002/npr2.12088

**Published:** 2019-11-27

**Authors:** Mami Okada‐Tsuchioka, Wataru Omori, Naoto Kajitani, Chiyo Shibasaki, Kei Itagaki, Minoru Takebayashi

**Affiliations:** ^1^ Division of Psychiatry and Neuroscience Institute for Clinical Research National Hospital Organization Kure Medical Center and Chugoku Cancer Center Kure Japan; ^2^ Department of Psychiatry National Hospital Organization Kure Medical Center and Chugoku Cancer Center Kure Japan; ^3^ Department of Neuropsychiatry Faculty of Life Sciences Kumamoto University Kumamoto Japan

**Keywords:** depression, electroconvulsive therapy, female‐specific, serum, thrombospondin‐1

## Abstract

**Aim:**

Thrombospondin‐1 (TSP‐1) is an astrocyte‐derived synaptogenesis‐related factor. It was previously reported to be increased by chronic treatment of electroconvulsive seizure, a model of electroconvulsive therapy (ECT), in rat hippocampus. The aim of this study was to examine whether serum levels of TSP‐1 are associated with depression and ECT.

**Methods:**

Serum TSP‐1 levels of major depressive disorder (MDD) patients (n = 36) and age‐ and gender‐matched healthy controls (n = 36) were measured by TSP‐1 ELISA. MDD patients were diagnosed according to the *Diagnostics and Statistical Manual of Mental Disorders*‐IV‐TR and underwent ECT. MDD patients were also analyzed for serum TSP‐1 levels pre‐ and post‐ECT. Evaluation of symptoms was obtained using the Hamilton Rating Scale for Depression.

**Results:**

Serum TSP‐1 levels showed significant decreases specific to female MDD patients. However, TSP‐1 did not change pre‐ and post‐ECT, did not correlate with symptoms, nor was not affected by the dose of antidepressants.

**Conclusion:**

Serum TSP‐1 is a possible female‐specific factor that reflects depressive trait, but not state.

## Abstract 2

The aim of this study was to examine whether serum levels of TSP‐1 are associated with depression and ECT. As a result, serum TSP‐1 levels showed significant decreases specific to female depressed patients. However, TSP‐1 did not change pre‐ and post‐ECT. In conclusion, serum TSP‐1 is a possible female‐specific factor that reflects depressive trait, but not state.

## Graphical abstract

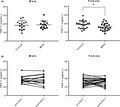

## 1 INTRODUCTION

Depression is a chronic debilitating illness affecting approximately 350 million people worldwide annually and imposes a major economic and medical burden on society. The pathology of depression has not been fully elucidated. Research with peripheral blood to find a biomarker for depression has been conducted; however, no objective biomarkers have yet to be found. As a possibility from various studies, abnormalities of synapses and astrocytes are suggested as being involved in the pathology of depression. For example, the reduction in synaptic contacts, the dysregulation of synapse‐related gene expression, and glial reduction have been found in several brain regions in depressed patients.^1^, ^2^, ^3^, ^4^, ^5^ Furthermore, the loss of astrocytes, the most abundant type of glia, is sufficient to induce depressive‐like behavior.^6^ Astrocytes tightly interact with synapses and engage in bidirectional communication critical to the processing of synaptic information. Therefore, a dysfunction of astrocytes is to contribute to the synaptic dysfunction present in depression.^7^ However, the common pathological molecule between synapse and astrocytes in depression has not yet been found.

Thrombospondin‐1 (TSP‐1) is a multifunctional extracellular matrix protein. It is rich in platelet alpha granules and was first discovered as a factor secreted by thrombin stimulation. In the peripheral system, several organs such as lung and bone express TSP‐1 and have been reported to be important for normal development and maintenance of homeostasis.^8^ TSP‐1 was also found to be secreted from platelets and other types of cells, such as endothelial cells, monocytes, neutrophils, macrophage, and adipocytes.^9^, ^10^, ^11^ It has various functions, including coagulation, antiangiogenesis, wound healing, and immune response.^12^ In the central nervous system (CNS), Christopherson et al reported that TSP‐1 is secreted by astrocytes, and promotes synaptogenesis, especially during presynaptic maturation in the developing brain.^13^ We previously reported that electroconvulsive seizure (ECS), an animal model of electroconvulsive therapy (ECT), significantly increased TSP‐1 mRNA and protein in rat hippocampus via the activation of astrocytes.^14^ Together with the above reports of synaptic and astrocytic abnormalities in depression, TSP‐1 is suggested as a possible target molecule of depression and its treatment in CNS. In this study, we investigated whether TSP‐1 in peripheral blood is possible as a biomarker of depression.

## 2 METHODS

### 2.1 Subjects

This study was conducted at the Department of Psychiatry of the National Hospital Organization Kure Medical Center between January 2011 and December 2017. Thirty‐six patients who were diagnosed according to *Diagnostics and Statistical Manual of Mental Disorders* (DSM)‐IV‐TR as major depressive disorder (MDD; 14 male and 22 female) were recruited among inpatients planning to receive ECT based on the guidelines of the American Psychiatric Association.^15^ ECT is often prescribed when a patient exhibits episodes of severe major depression, psychosis, and catatonia or has shown insufficient improvement with prescribed pharmacotherapy treatment.^15^

Patients with a past or present history of substance abuse, substance dependence, significant neurological illness, or any other significant medical illness were excluded from participation in the current study. After procedures were fully explained, written informed consent was obtained from all subjects to participate in the study. Thirty‐six age‐ and gender‐matched serum samples (14 male and 22 female) with no history of past or current psychiatric disorders were picked from among previously collected blood from the healthy subject blood bank owned by the Kure Medical Center, as healthy control subjects. The current study was approved by the Ethics Committee of the National Hospital Organization Kure Medical Center.

### 2.2 ECT procedure

ECT was performed according to procedures of a previous report.^16^ Anesthesia was induced with thiamylal sodium (2‐3 mg/kg, iv) and suxamethonium chloride (0.5‐1 mg/kg, iv). The ECT device used was the Thymatron System IV brief pulse square‐wave apparatus (Somatics Inc). Because there are many highly urgent and severe cases, electrodes were positioned bilaterally on the frontal‐temporal region. Only one adequate seizure was required for each session, which was defined as an electroencephalographic seizure persisting more than 25 seconds with a high amplitude, slow wave, and postictal suppression. The initial stimulus dose was determined using the half‐age method.^17^ If an adequate electroencephalographic seizure occurred in one session, the same stimulus energy was used in the next session. When a missed or an inadequate seizure occurred, the patient was restimulated with 1.5‐2 times the initial stimulus. The maximum number of stimulations for each treatment session was 2. ECT was administered a maximum of three times per week. If any adverse effects (eg, cognitive dysfunction and delirium) occurred, the frequency of the ECT schedule was reduced to once or twice per week. ECT continued until the patient was asymptomatic or the attending psychiatrist determined that the patient had obtained the maximum benefit.

### 2.3 Assessment of clinical symptoms

Clinical symptomatic scores were assessed by three trained psychiatrists using the 17‐item Hamilton Rating Scale for Depression (HAM‐D) prior to the first ECT session (pre‐ECT) and after the final ECT session (post‐ECT).

### 2.4 TSP‐1 ELISA

Venous blood samples were taken in the morning (between 7:00 and 8:00 am) at pre‐ECT and post‐ECT. Blood samples were drawn into anticoagulant‐free tubes, kept at room temperature for 30 minutes, and serum was separated by centrifugation at 1500 g for 15 minutes at 4°C. Serum samples were stored at −80°C until assay. Serum TSP‐1 levels were measured using TSP‐1 ELISA (Quantikine ELISA, R&D Systems) according to the manufacturer's instructions.

### 2.5 Statistical analysis

The data are presented as the mean ± SD or median and interquartile ranges. The normal distribution of data was examined by the Shapiro‐Wilk test. Significant differences between two groups were evaluated by using Student's *t* test for the normal distribution data or Mann‐Whitney *U* test for the non‐normal distribution data. The receiver operating characteristic (ROC) curve for the specificity and the sensitivity of serum TSP‐1 was calculated to discriminate between the MDD and control. The area under the ROC curve (AUC) was calculated for serum TSP‐1 as well (AUC = 0.5 indicates no discrimination, and AUC = 1 would indicate a perfect diagnostics test). A Wilcoxon signed‐rank test was used to evaluate the statistical differences in the parameters between pre‐ECT and post‐ECT. The association between various clinical factors of MDD patients, such as gender, age at ECT, age at onset, number of ECT sessions per course, imipramine (IMI)‐equivalent dose at pre‐ECT, platelet counts, and serum TSP‐1 levels, was firstly analyzed by univariate linear regression analysis. Subsequently, multivariate linear regression analysis with stepwise method was conducted in order to correct for confounding factors. The relationship between body mass index (BMI) and serum TSP‐1 of all subjects was analyzed using univariate linear regression analysis by gender. Statistical significance was defined as *P* < .05. Analyses were performed by using SPSS version 22.0 for Windows (IBM Japan Corporation).

## 3 RESULTS

**Figure 1:**
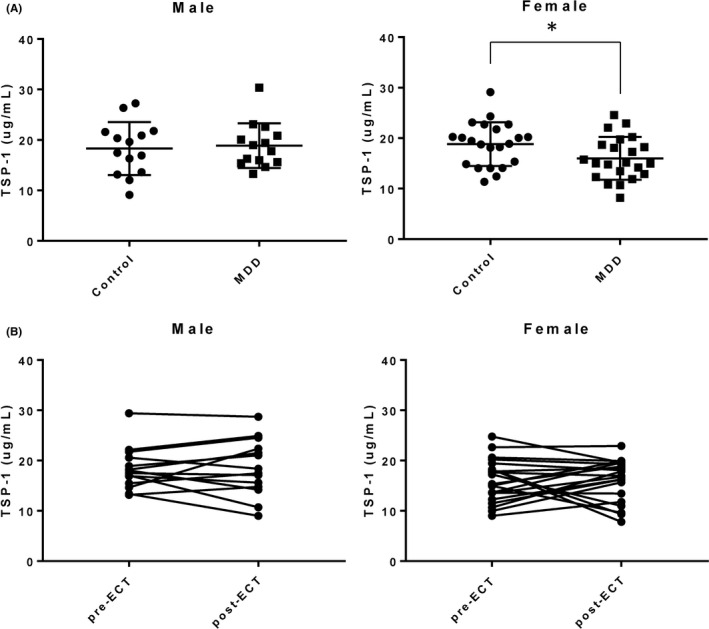
A, Comparison of serum TSP‐1 concentrations between controls and MDD patients separately by gender. Data were shown as the mean ± SD. *P < .05, compared with female controls (Student's *t* test). B, Comparison in serum TSP‐1 concentrations between pre‐ and post‐ECT separately by gender (Wilcoxon signed‐rank test)

Serum TSP‐1 levels of controls and MDD patients were first compared, and there was no statistical difference (control: 18.62 ± 4.65 μg/mL, MDD: 17.12 ± 4.50 μg/mL, *P* = .167). Then, the levels of serum TSP‐1 were compared by gender. The serum TSP‐1 level was significantly lower in female MDD patients (MDD‐F) than female controls (Cont‐F). Meanwhile, there was no difference in male MDD patients (MDD‐M) and male controls (Cont‐M; Figure 1A). In the ROC curve analysis of diagnosis, the AUC of serum TSP‐1 in females was 0.678, suggesting that serum levels of TSP‐1 may be of low accuracy to diagnose MDD in females.

As shown in Table 1, HAM‐D scores were significantly decreased after the course of ECT session, thus indicating that depressive symptoms were significantly improved by ECT. However, no changes in serum TSP‐1 levels were observed between pre‐ and post‐ECT (Figure 1B). Consistent with these results, depressive symptoms of pre‐ and post‐ECT were not predictive for serum TSP‐1 levels in MDD patients (nonstandardized coefficient *Β *= −0.067 ± 0.048, *P* = .168).

**Table 1:** Subjects’ demographic data

	Male			Female			Sex difference
Factor	Control	MDD	*P*‐value	Control	MDD	*P*‐value	*P*‐value^a^
Number	14	14		22	22		
Age at ECT (y)	56.1 ± 12.8	57.7 ± 12.0	.740^b^	59.3 ± 13.6	59.8 ± 13.8	.913^b^	.649
BMI (kg/m^2^)	23.6 ± 2.6	21.5 ± 3.4	.095^b^	22.7 ± 3.1	21.1 ± 3.8	.140^b^	.748
Age at onset (y)	—	55.4 ± 13.7		—	53.6 ± 13.7		.705
Number of ECT sessions per course	—	12.0 [11.0‐12.3]		—	10.5 [6.8‐12.3]		.116
HAM‐D score at pre‐ECT	—	21.0 [16.5‐32.5]		—	21.0 [17.8‐29.0]		.820
HAM‐D score at post‐ECT	—	4.0 [2.8‐7.0]	.001^c^	—	4.0 [2.5‐6.5]	<.001^c^	.803
IMI‐equivalent dose at pre‐ECT (mg/d)	—	136.6 ± 121.3		—	244.9 ± 172.1		.048
Platelet counts (×10^4^)	—	23.9 [20.4‐27.5]		—	24.2 [21.3‐27.0]		.810

Notes

Data are shown as the mean ± SD or median [interquartile ranges].

Abbreviations: BMI, body mass index; ECT, electroconvulsive therapy; HAM‐D, Hamilton Rating Scale for Depression; IMI, imipramine; MDD, major depressive disorder.

a *P*‐values of comparison between male and female MDD by Student's *t* test or Mann‐Whitney *U* test.

b *P*‐values of comparison between control and MDD by Student's *t* test.

c *P*‐values of comparison between scores at pre‐ECT and those at post‐ECT by Wilcoxon signed‐rank test.

Subsequently, the influence of clinical factors such as gender, age at ECT, age at onset, the number of ECT sessions per course, IMI‐equivalent dose at pre‐ECT, and platelet counts was analyzed. First, a univariate linear regression analysis was done with individual clinical factors. Reflecting that platelets are known as the main storage location of TSP‐1 in serum, the serum TSP‐1 levels were significantly predicted by platelet counts. Age at onset was also a significant predictive factor of serum TSP‐1. In addition, gender and IMI‐equivalent dose tended to predict serum TSP‐1 levels (*P* < .1; Table 2). In order to correct for confounding factors, these factors were subsequently analyzed by multivariate linear regression analysis with stepwise method. As a result, platelet counts as well as gender entered the regression model and were revealed as significant predictive factors of serum TSP‐1 levels in MDD (Table 3). To summarize these results, the serum TSP‐1 levels of MDD patients were not affected only by the platelet counts, and have a gender dependency. In other words, serum TSP‐1 is significantly lower in MDD‐F than MDD‐M after correcting the platelet counts.

**Table 2:** Univariate linear regression analysis of individual factors for serum TSP‐1 in MDD

	*R* ^2^	Constant	95% CI	Nonstandardized coefficient *Β*	95% CI	*P*‐value
Gender (base: male)	.101	18.881	16.532 to 21.231	−2.890	−5.895 to 0.115	.059
Age at ECT	.067	22.419	15.381 to 29.456	−0.090	−0.207 to −0.027	.126
Age at onset	.160	24.319	18.393 to 30.245	−0.133	−0.238 to −0.027	.016*
Number of ECT sessions per course	.042	14.289	9.330 to 19.248	0.271	−0.182 to 0.725	.232
IMI‐equivalent dose at pre‐ECT	.081	18.724	16.327 to 21.121	−0.008	−0.017 to −0.001	.092
Platelet counts	.340	4.476	−1.787 to 10.739	0.510	0.262 to 0.757	<.001*

Abbreviations: ECT, electroconvulsive therapy; IMI, imipramine; MDD, major depressive disorder; TSP‐1, thrombospondin‐1.*P<.05.

**Table 3:** Multivariate linear regression analysis for serum TSP‐1 in MDD

	Nonstandardized coefficient *Β*	95% CI	*P*‐value
Constant	6.153	0.191 to 12.114	.043*
Gender (base: male)	−3.013	−5.403 to −0.623	.015*
Platelet counts	0.516	0.287 to 0.746	<.001*

Note

Two variables were selected among six variables by stepwise method.

*R* ^2 ^= .450, model *P* < .001.

Abbreviations: MDD, major depressive disorder; TSP‐1, thrombospondin‐1.*P<.05.

There was a significant difference between the IMI‐equivalent dose at pre‐ECT in MDD‐M and MDD‐F (Table 1). However, the IMI‐equivalent dose at pre‐ECT was not extracted as a significant predictive factor as a result of multivariate linear regression analysis (Table 3). Therefore, the doses of antidepressant may not affect the lower serum TSP‐1 levels in MDD‐F in this study.

A previous study indicated a relationship between BMI and serum TSP‐1 specifically in females.^18^ However, there was no significant difference in BMI between Cont‐F and MDD‐F (Table 1). Furthermore, BMI did not predict serum TSP‐1 levels in females (nonstandardized coefficient *Β* = 0.074, *P* = .71). Therefore, the significant difference in TSP‐1 levels in females seen in this study was not attributed to BMI.

## 4 DISCUSSION

The main finding of this study is as follows: (a) Serum TSP‐1 levels showed a specific decrease in MDD‐F, but at an insufficient level for diagnostic ability; (b) serum TSP‐1 levels did not change pre‐ and post‐ECT; and (c) serum TSP‐1 levels were not correlated with depressive symptoms or doses of antidepressants. In conclusion, serum TSP‐1 is a possible female‐specific factor that reflects depressive traits, but not state.

We previously reported that chronic ECS increased TSP‐1 mRNA and protein in the hippocampus of rats.^14^ From the perspective of translational research, we expanded to research with human samples. As a preliminary investigation, TSP‐1 levels in cerebrospinal fluid (CSF) of several control subjects were tried to investigate. However, CSF TSP‐1 levels were less than the detection limit of TSP‐1 ELISA (0.944 ng/mL) used in this study. Thus, TSP‐1 levels in CSF are expected to be very low. Therefore, the investigation with CSF was thought to be difficult to evaluate TSP‐1 levels. Thus, we examined using peripheral blood in this study.

Contrary to the previous results of ECS in rat hippocampus, ECT had no effect on serum TSP‐1 levels in humans in the present study. Regarding the discrepancy of these results, the difference in the samples is considered to be greatly affected. In the previous study, the expression of TSP‐1 extracted from hippocampal tissue was evaluated, and thus, the changes in the hippocampus could be captured. On the other hand, serum, a component of body fluid, was used in this study. Serum contains a large amount of platelet‐derived TSP‐1 and might be affected by various peripheral organs that express TSP‐1. There is no study showing the relationship between serum TSP‐1 levels and CNS TSP‐1 levels. Since serum contains TSP‐1 not derived from CNS, the alteration by ECT in CNS might not be detectable. In order to analyze the function of TSP‐1 in the CNS precisely, further studies are required to analyze the expression in specific brain regions using the brain tissue of a depression model or postmortem human brains.

A previous study indicated a relationship between BMI and serum TSP‐1 specifically in females. Serum TSP‐1 levels showed a significant positive correlation with BMI in females.^18^ Thus, we also investigated whether BMI affected the TSP‐1 levels of MDD‐F in this study. It revealed that BMI did not affect serum TSP‐1 levels. The discrepancy between this study and previous reports may be due to differences in subject characteristics. That is, there were few BMI >25 subjects in this study (3%) compared with the previous study (60%). Therefore, there is a possibility that serum TSP‐1 was significantly decreased in MDD‐F due to several factors other than BMI. Platelets were also investigated as a potential factor that affects serum TSP‐1. However, the multivariate linear regression analysis shows that there seem to be other affecting factors besides platelet counts.

Epidemiologically, it has been reported that females are more likely to be depressed at a rate about twice as often as males.^19^ Depression which is clinically attributed to estrogen deficiency is often experienced, for example, depression after ovariectomy, postmenopausal, and due to antiestrogen drugs, such as tamoxifen. Taking these things into consideration, it is assumed that female hormones, mainly estrogen, tend to decrease in female depression, and as a result, serum TSP‐1 decreases. Although several reports showed that the deficiency or reduction in female hormone increased TSP‐1 in various types of tissues,^20^, ^21^, ^22^ some contrary reports prove the opposite direction.^23^ Therefore, there is a possibility that female hormones may have an effect. It is necessary to further investigate the involvement of female hormones and the extent of such effects on serum TSP‐1 levels.

This study had some limitations. The sample size was small, and thus, it is desirable to increase the number of subjects, preferably extending to cohort research. All depressed patients in this study are taking medications. Although IMI‐equivalent dose was not predictive with TSP‐1 levels, it is more desirable to examine in the serum of drug‐naive patients. Platelet counts of controls were not obtained because the serum samples were from blood samples already collected at our own biobank. Thus, the comparison between Cont‐F and MDD‐F was conducted without correcting platelet counts. The value of female hormones was not measured in the samples of this study. Therefore, further study should be considered that includes female hormone levels and platelet counts.

Serum TSP‐1 levels were significantly reduced in MDD‐F; however, it did not change pre‐ and post‐ECT. The diagnostic ability was insufficient level. Therefore, serum TSP‐1 could not be a diagnostic marker for depression. Since serum TSP‐1 was consistently reduced in MDD‐F in a state‐independent manner, we consider that it is possible to be a female‐specific trait factor of depression. It may be used as a factor to assess the predisposition of depression in females. Further investigation is necessary with consideration of the limitation in this study. Alternatively, serum TSP‐1 alone is difficult to diagnose depression with single biomarker, but it may possible to determine in the future by combining several biomarkers.

## CONFLICT OF INTEREST

The authors declare no conflict of interest.

## AUTHOR CONTRIBUTIONS

MOT conducted the experiments, analyzed the results, and wrote the initial draft of the manuscript. WO, KI, and CS collected the samples. NK and WO performed the experimental support, analyzed the data, and prepared the figure and tables. All authors contributed to the discussion. MT designed the experiments and revised the manuscript. All authors reviewed the results and approved the final version of the manuscript.

## DATA REPOSITORY

We agree to deposit the data and publish part of it as supporting information.

## APPROVAL OF THE RESEARCH PROTOCOL BY AN INSTITUTIONAL REVIEWER BOARD

The protocol for this research project has been approved by a suitably constituted Ethics Committee of the National Hospital Organization Kure Medical Center (Approval No. 26‐52, 27‐09).

## INFORMED CONSENT

All informed consent was obtained from the subjects to participate in the study.
